# Design of Trabecular Bone-Inspired Mechano-Acoustic Coupling Porous Structures

**DOI:** 10.3390/ma19122603

**Published:** 2026-06-17

**Authors:** Yiyan Lin, Jundong Zhang, Chaolei Zhang, Ruiyao Liu, Zhenglei Yu

**Affiliations:** 1 College of Biological and Agricultural Engineering, Jilin University, Changchun 130022, China; 2 Central Research Institute, China Automotive Technology and Research Center Co., Ltd., Tianjin 300300, China; 3 School of Mechanical and Aerospace Engineering, Jilin University, Changchun 130025, China

**Keywords:** bionic design, additive manufacturing, mechano-acoustic coupling, implicit surface structures

## Abstract

Aiming at the technical bottleneck that traditional porous structures can hardly achieve mechanical load-bearing and acoustic regulation simultaneously, this study designs and fabricates three implicit surface porous structures (Gyroid, Diamond, Lidinoid) based on the bionic principle of trabecular bone. Experimental characterization and numerical analysis of their mechano-acoustic coupling performance are systematically carried out. Selective Laser Melting (SLM) technology is employed to realize the integrated forming of 316L bionic structures. Quasi-static compression experiments and finite element simulations are conducted to reveal the progressive deformation mechanism and energy absorption characteristics of different topological configurations. The results indicate that the Diamond structure exhibits the optimal comprehensive performance in terms of load-bearing capacity, specific energy absorption and isotropy. On this basis, the sound absorption and sound insulation performances of the structures are evaluated via an acoustic impedance tube test. The results show that the Diamond structure possesses a remarkably higher sound absorption coefficient and sound insulation value in the high-frequency range than other configurations, demonstrating excellent acoustic energy dissipation and sound wave isolation capability. The research indicates that the synergistic optimization of mechanical and acoustic performances can be achieved by regulating the Triply Periodic Minimal Surface (TPMS) topological configuration. Benefiting from its efficient stress transfer paths and intricate sound wave propagation channels, the Diamond structure realizes the coupling of high load-bearing capacity, superior energy absorption and favorable acoustic performance. This work provides a theoretical basis and technical support for the design of bionic porous structures in multifunctional scenarios such as bone implants and protective noise reduction.

## 1. Introduction

With the escalating demand for structural performance in aerospace, rail transit, biomedical implants and other fields [1,2,3,4,5], traditional single-function porous materials can hardly meet the multiple requirements of high load-bearing protection, efficient energy absorption and noise control simultaneously. The development of multifunctional structures with excellent mechanical properties and acoustic regulation capability has become an important research direction in the fields of materials science and mechanical engineering [6,7,8,9,10]. Biological tissues such as natural trabecular bone achieve the collaborative optimization of mechanical load-bearing and mass transport through hierarchical and multi-scale porous topology, providing significant bionic inspiration for the design of artificial multifunctional structures [11,12,13,14,15]. Among them, Triply Periodic Minimal Surface (TPMS) structures constructed based on implicit surfaces are regarded as ideal carriers for the synergistic regulation of mechanical and acoustic performances. Featuring continuously smooth pore channel topology, high specific surface area, excellent mechanical designability and uniform stress distribution characteristics, TPMS structures have become a research hotspot in the field of bionic porous structures in recent years [16,17,18,19,20].

In terms of the mechanical properties of TPMS porous structures, scholars have conducted extensive fundamental research. For instance, Wan et al. [21] compared the quasi-static compressive responses of four typical TPMS topologies including Gyroid and Diamond through experimental and numerical methods. The results revealed that the Diamond structure possesses higher load-bearing capacity and energy absorption efficiency due to its highly symmetric truss-like topology. She et al. [22] further investigated the influence of unit morphological characteristics on the mechanical performance of TPMS structures, and established a correlation model between topological morphological parameters and mechanical properties. Moreover, Daynes [23] demonstrated that the continuous surface characteristics of TPMS structures can effectively improve their mechanical functional performance and remarkably enhance structural mechanical stability. These studies have laid an important foundation for understanding the mechanical behavior of TPMS structures. However, most existing studies mainly focus on the characterization of a single mechanical property, while the complex multi-field coupling working conditions faced by structures in practical engineering applications are rarely fully considered. In terms of acoustic performance research, the unique pore channel characteristics of TPMS porous structures have also attracted widespread attention. Sirivuri et al. [24] explored the effects of different TPMS configurations on sound absorption performance, and found that the continuous curved pore channels of TPMS structures can prolong the propagation path of sound waves and strengthen the viscous dissipation effect of air, thereby improving sound absorption efficiency. Chua et al. [25] proposed a novel multi-scale acoustic framework to compare the acoustic performance of TPMS topologies, providing a methodological basis for the design and optimization of structural acoustic properties. In addition, several studies have attempted to realize acoustic regulation in specific frequency bands via topology optimization. Nevertheless, most designs only target a single acoustic performance without integrating the synergistic optimization of mechanical and acoustic properties.

Although existing studies have made certain progress in investigating the mechanical or acoustic properties of TPMS structures separately, most of them only analyze a single mechanical or acoustic performance [26,27]. There is a lack of systematic characterization on the coupled mechano-acoustic properties of the same topology, making it difficult to evaluate the comprehensive performance of TPMS structures in multifunctional application scenarios. Moreover, the majority of current research focuses merely on a single material or a single working condition, and fails to carry out full-process design and verification combined with the manufacturability of additive manufacturing processes, which greatly limits their potential for practical engineering applications [28,29,30].

Based on the above research background, this study designs and fabricates three typical TPMS porous structures, namely Gyroid, Diamond and Lidinoid, inspired by the bionic concept of natural trabecular bone. Selective Laser Melting (SLM) additive manufacturing technology is adopted to realize the integrated forming of 316L bionic structures. Quasi-static compression experiments and finite element simulations are conducted to systematically characterize the load-bearing capacity, energy absorption characteristics and progressive deformation and failure modes of different topological configurations. Meanwhile, sound impedance tube tests are performed to evaluate the sound absorption and sound insulation performances of the structures in a broadband frequency range. This study carries out full-process structural design and performance verification considering the manufacturability of additive manufacturing, and conducts integrated mechano-acoustic experiments and coupled finite element characterization. By exploring the inherent law that TPMS topological configurations dominate the load-bearing, energy absorption and noise reduction performances, this work aims to reveal the mechano-acoustic coupling regulation mechanism of different TPMS bionic porous structures, and further provide a solid theoretical basis and feasible technical support for the optimal design of bionic porous structures applicable to multifunctional scenarios including aerospace protection and biomedical implants.

## 2. Materials and Methods

### 2.1. Femur-Inspired Bionic Structural Design and Fabrication

**Figure 1:**
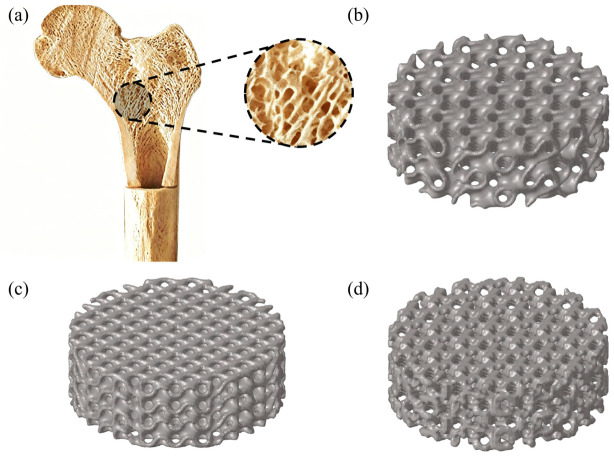
Design of TPMS porous structures inspired by femoral cancellous bone. (**a**) Macro morphology of the proximal human femur and the micro trabecular network of its cancellous bone; (**b**) Gyroid-type TPMS porous structure; (**c**) Diamond-type TPMS porous structure; (**d**) Lidinoid-type TPMS porous structure.

This design takes the porous microstructure of the proximal femoral cancellous bone as the bionic prototype. Proximal femoral cancellous bone is composed of highly ordered three-dimensional trabecular networks, featuring a hierarchical porous structure, high specific strength, excellent stress conduction and energy absorption characteristics, and serving as a naturally efficient lightweight load-bearing structure. Figure 1a clearly shows the macro morphology of the proximal femur and the micro trabecular network in its cancellous bone region, whose porosity, connectivity and topological structure provide dual biomechanical and biological references for the design of porous implants.

Based on the bionic design concept of femoral cancellous bone, three typical TPMS porous structures, namely Gyroid, Diamond and Lidinoid, are constructed using nTopology 5.9.2 software, as shown in Figure 1b–d. TPMS structures possess zero mean curvature, high connectivity, tunable porosity and topological uniformity, and can effectively replicate the mechanical and biological characteristics of trabecular bone. The Gyroid structure exhibits continuously smooth curved features with helically three-dimensionally interconnected pore channels and no sharp corners. The Diamond structure is based on the topological configuration of Diamond crystals and is characterized by high symmetry and uniform pore distribution. The Lidinoid structure integrates high connectivity and complex pore topology with a larger specific surface area and superior potential for graded pore regulation. All three TPMS structures inherit the bionic design philosophy of cancellous bone, and customized design of porosity, pore size and topological morphology can be realized through parametric regulation.

**Figure 2:**
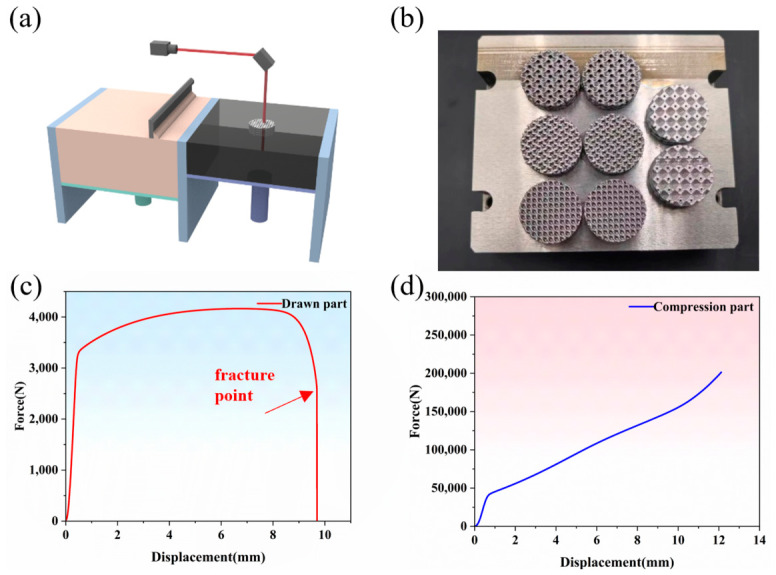
Fabrication and mechanical property testing of 316L bionic porous structures. (**a**) Schematic diagram of the Selective Laser Melting (SLM) additive manufacturing process for bionic porous structures; (**b**) physical photographs of bionic porous specimens with different topological configurations fabricated by SLM process; (**c**) quasi-static tensile force–displacement curve of 316L bulk material; (**d**) quasi-static compressive force–displacement curve of solid 316L cylindrical specimen.

In this study, the Selective Laser Melting (SLM) additive manufacturing process is adopted to realize the integrated forming of bionic TPMS porous structures, and the principle of the forming process is illustrated in Figure 2a. High-precision forming of complex curved topological structures is achieved by precisely controlling the printing parameters, including a laser power of 180 W, a scanning spacing of 100 μm, a scanning speed of 900 mm/s, and a layer thickness of 20 μm. The designed printed structure has a diameter of 29 mm and a height of 10 mm, and the physical specimens of the three configurations are presented in Figure 2b. Quasi-static tensile tests are carried out on 316L bulk specimens, and the corresponding force–displacement curves are shown in Figure 2c, which exhibit typical characteristics of plastic deformation and necking fracture. To clarify the intrinsic mechanical properties of the material, quasi-static compressive tests are performed on solid 316L cylindrical specimens, and their force–displacement curves are displayed in Figure 2d. The curves reflect the elastoplastic deformation and yield hardening behavior of the material under axial compressive loading, providing an accurate material constitutive basis for the subsequent mechanical analysis and numerical simulation of porous structures. This also comprehensively reveals the differences in mechanical behavior of the material under tensile and compressive loads.

### 2.2. Femur-Inspired Bionic Structural Experimental and Numerical Setup

**Figure 3:**
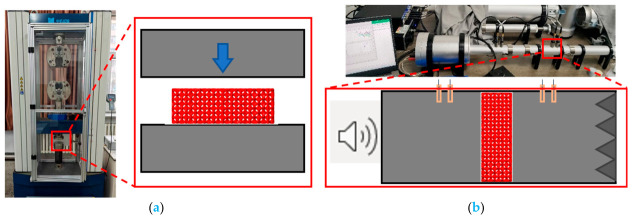
Performance test apparatus for bionic TPMS porous structures. (**a**) Universal material testing machine and schematic diagram of specimen loading; (**b**) acoustic impedance tube experimental setup and schematic diagram of specimen installation.

To evaluate the static load-bearing capacity of porous structures, a universal material testing machine (Model ZDSHPB-4080, Zongde, Taian, Shandong, China) is employed to conduct quasi-static compression tests. The test apparatus and specimen loading configuration are shown in Figure 3a. Axial loading is applied at a constant rate of 4 mm/min to obtain the force–displacement response of the structure under low strain rate conditions, which provides an experimental basis for analyzing its load-bearing and energy absorption characteristics. To investigate the acoustic performance of bionic porous structures, an acoustic impedance tube testing system (Model BSWA SW, BSWA Technology, Beijing, China) is established. The experimental setup and specimen installation scheme are illustrated in Figure 3b. Based on the standing wave method, sound waves are excited inside the impedance tube and sound pressure signals are collected to measure the sound absorption coefficient and sound insulation properties of porous specimens. This study reveals the regulation mechanism of different TPMS topologies on sound wave propagation, and provides performance support for their application in noise reduction scenarios. Furthermore, to guarantee the reliability and accuracy of experimental data, all tests in this study were repeated three times, and the average values were taken as the final experimental results for subsequent analysis.

**Figure 4:**
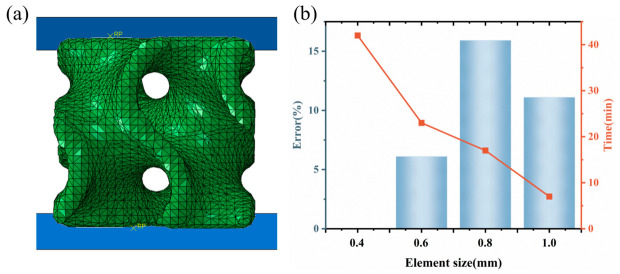
Mesh independence verification of bionic TPMS porous structures. (**a**) Simulation settings of Diamond-type unit cell structure; (**b**) error vs. time for different mesh sizes.

In this study, quasi-static compression simulation of bionic porous structures was performed using Abaqus2023 software to thoroughly explore the mechanical behaviors of bionic skeleton structures under compressive loading. The boundary conditions were established based on the quasi-static compression experimental scheme. The bottom surface of the bionic skeleton structure was fully constrained to limit all translational and rotational degrees of freedom in the x, y, and z directions, maintaining a fixed boundary state throughout the loading process. A uniform displacement load in the negative y-axis direction was applied to the top surface of the structure, as depicted in Figure 4a. In terms of contact settings, general contact was adopted between the upper and lower surfaces of the structure and the loading plates. This contact configuration accurately defines the interfacial interaction and ensures the effective transmission of normal pressure on the contact surface.

The C3D10M ten-node modified quadratic tetrahedral element, which is compatible with the implicit solving algorithm, was selected for mesh discretization. Considering the high computational cost of full-scale structural simulation and to improve analytical efficiency, a single unit cell of the Diamond structure was adopted as the simulation object. The unit cell model was meshed in HyperMesh 2021 with four element sizes of 0.4 mm, 0.6 mm, 0.8 mm, and 1.0 mm. Taking the simulation results of the 0.4 mm mesh as the benchmark, mesh independence verification was completed by comparing the mechanical responses obtained under different mesh sizes. The corresponding verification results are displayed in Figure 4b.

## 3. Results and Analysis of Structural Mechanical Properties

**Figure 5:**
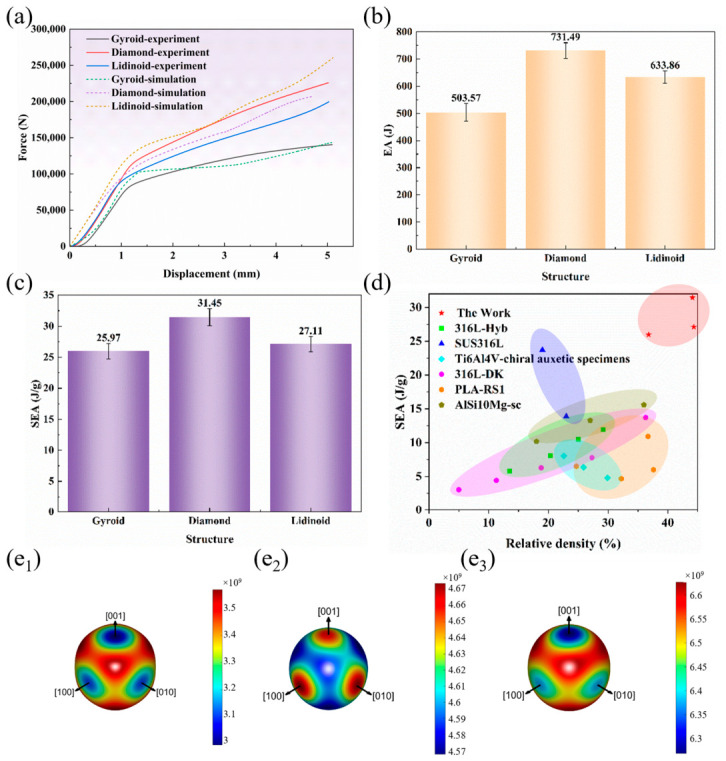
Quasi-static compressive mechanical properties of bionic TPMS porous structures. (**a**) Comparison of experimental and simulated force–displacement curves; (**b**) comparison of energy absorption (EA) performance; (**c**) comparison of specific energy absorption (SEA) performance; (**d**) Ashby chart of SEA versus relative density for the proposed structures and several typical lattice structures; (**e_1_**–**e_3_**) polar diagrams of elastic anisotropy for unit cells of the three structures.

The quasi-static compressive mechanical properties of three bionic TPMS porous structures are characterized, and the results are presented in Figure 5. Among them, Figure 5a compares the experimental and simulated force–displacement curves of Gyroid, Diamond and Lidinoid topologies. The results indicate that the simulated curves exhibit a highly consistent variation trend with the experimental curves in both the elastic stage and yield plateau stage, which verifies that the established numerical model can accurately reproduce the mechanical response and deformation behavior of porous structures. In terms of load-bearing capacity, the peak load of the three structures shows obvious differences. The Diamond structure possesses the optimal load-bearing capacity, followed by the Lidinoid structure, while the Gyroid structure exhibits the lowest capacity. Such differences originate from the spatial stress transfer paths of different topological configurations. The Diamond structure achieves efficient stress transfer through a highly symmetric truss-like distribution, whereas the helical curved features of the Gyroid structure easily induce premature local stress concentration, thereby reducing the overall load-bearing capacity.

Further analysis of energy absorption performance shows in Figure 5b that the total energy absorption (EA) value of the Diamond structure reaches 731.49 J, which is significantly higher than 633.86 J of the Lidinoid structure and 503.57 J of the Gyroid structure. The comparison results of specific energy absorption (SEA) in Figure 5c follow the same trend. The SEA value of the Diamond structure is 31.45 J/g, superior to 27.11 J/g of the Lidinoid structure and 25.97 J/g of the Gyroid structure. This demonstrates that the Diamond structure not only has a stronger total energy dissipation capability, but also achieves higher energy absorption efficiency under lightweight conditions, making it more suitable for scenarios requiring both light weight and impact resistance, such as aerospace protection and orthopedic implants. To further evaluate its performance advantages, Figure 5d compares the SEA-relative density performance of the three TPMS structures in this study with various porous materials reported [31,32,33,34,35,36]. The results show that the proposed structures still deliver remarkably higher SEA values than other materials at a relatively high relative density, locating in the superior region of the performance distribution. It is confirmed that the continuously smooth curved surface and uniform stress distribution characteristics of TPMS topologies effectively avoid the stress concentration problem of traditional truss structures and achieve a prominent improvement in energy absorption efficiency. Finally, the elastic anisotropy analysis (Figure 5e_1_–e_3_) reveals differences in the elastic modulus distribution among the three structures. The Diamond structure shows the most uniform modulus distribution and presents nearly isotropic characteristics, while the Gyroid and Lidinoid structures exhibit an obvious modulus gradient along different crystallographic directions. Specifically, the modulus is higher in the [001] direction and relatively lower in the [100] and [010] directions. This anisotropy difference provides a direct basis for structural application selection. For scenarios with high requirements on mechanical isotropy such as orthopedic implants, the Diamond structure is more conducive to achieving mechanical matching with natural bone tissue and reducing the stress shielding effect. For protective scenarios without special isotropy requirements, the directional dependence of Gyroid and Lidinoid structures can also be utilized by optimizing the loading direction.

**Figure 6:**
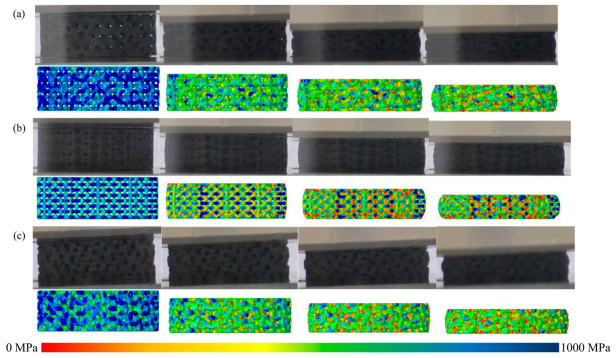
Deformation and stress evolution of bionic TPMS porous structures under quasi-static compression. (**a**) Experimental deformation and equivalent stress contour cloud of Gyroid structure at compressive displacements ranging from 0 to 5 mm; (**b**) experimental deformation and equivalent stress contour cloud of Diamond structure at compressive displacements ranging from 0 to 5 mm; (**c**) experimental deformation and equivalent stress contour cloud of Lidinoid structure at compressive displacements ranging from 0 to 5 mm.

By means of quasi-static compression experiments and finite element simulations, the progressive deformation behavior and stress evolution law of three bionic TPMS porous structures, namely Gyroid, Diamond and Lidinoid, are comparatively analyzed. From left to right, the four groups of images sequentially present the experimental deformation photographs and equivalent stress contours at different compression stages. The results show that all three structures exhibit the typical deformation process of porous materials, including elastic deformation, yield collapse and densification. Nevertheless, topological configuration significantly regulates their failure modes and stress distribution characteristics. As shown in Figure 6a, the Gyroid structure achieves more uniform stress benefiting from its continuously smooth curved topology. No obvious local shear band appears during compression, and the structure undergoes a layer-by-layer progressive collapse failure mode, in which the stress concentration effect is effectively suppressed. In Figure 6b, the highly symmetric truss-like topology of the Diamond structure endows it with superior load-bearing capacity, but also induces more prominent early-stage stress concentration. Local high-stress bands tend to form and further trigger shear-dominated failure. In Figure 6c, the failure behavior of the Lidinoid structure lies between the above two, presenting a mixed characteristic of uniform deformation and local buckling. Meanwhile, the high-stress regions predicted by finite element stress contours are in good agreement with the positions where buckling and collapse occur in experimental specimens. This verifies the accurate prediction capability of the numerical model for structural deformation behavior and failure modes, and further reveals the regulation mechanism of TPMS topological configuration on the mechanical response and energy absorption characteristics of porous structures.

## 4. Results and Analysis of Structural Acoustic Properties

**Figure 7:**
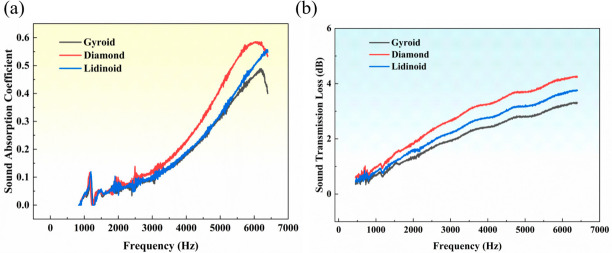
Acoustic performance of bionic TPMS porous structures. (**a**) Variation curves of sound absorption coefficient with frequency for different topological structures; (**b**) variation curves of sound insulation loss with frequency for different topological structures.

The acoustic performances of three bionic TPMS porous structures (Gyroid, Diamond, Lidinoid) are systematically characterized, and the results are shown in Figure 7. In terms of sound absorption performance (Figure 7a), the sound absorption coefficients of all three structures increase significantly with the rise in frequency, showing the typical sound absorption characteristics of porous materials. Among them, the Diamond structure exhibits the optimal sound absorption performance, with a peak sound absorption coefficient close to 0.6 in the high-frequency range (approximately 6000 Hz), followed by the Lidinoid structure, while the Gyroid structure performs relatively poorly. This indicates that topological configuration exerts a remarkable regulatory effect on the viscous dissipation and resonance effect of sound waves. The sound insulation test results (Figure 7b) follow a consistent trend with the sound absorption performance. The Diamond structure achieves the highest sound insulation value, reaching approximately 4.3 dB at 6000 Hz, followed by the Lidinoid structure, and the Gyroid structure has the lowest value. It demonstrates that the Diamond topology not only dissipates acoustic energy more effectively, but also improves the sound insulation effect through more complex sound wave reflection and scattering paths. Overall, all three TPMS structures present excellent potential for acoustic regulation in the high-frequency range. Benefiting from its unique pore channel structure and sound wave propagation paths, the Diamond structure shows obvious advantages in both sound absorption and sound insulation performances. Although the specimens achieve optimal sound absorption and insulation at 6 kHz, high-frequency noise commonly exists in aerospace and rail transit equipment, which provides performance support for their engineering application in noise reduction scenarios. The comparative analysis of mechanical and acoustic properties of the bionic porous structures is presented in Table 1.

## 5. Conclusions

Based on the bionic concept of trabecular bone, three implicit surface TPMS porous structures are designed and fabricated. The regulation law of topological configuration on their mechano-acoustic coupling performance is systematically revealed, and the multifunctional collaborative optimization of mechanical and acoustic properties is realized. The main conclusions are drawn as follows:

(1) Topological configuration significantly regulates the quasi-static mechanical behavior and failure modes of TPMS porous structures. All three structures exhibit typical compressive response characteristics of porous materials. Among them, the Diamond structure possesses the highest load-bearing capacity, with the optimal total energy absorption (731.49 J) and specific energy absorption (31.45 J/g), as well as the highest degree of mechanical isotropy. The Gyroid structure shows uniform stress distribution and presents a stable progressive layer-by-layer collapse failure mode. The mechanical properties and failure characteristics of the Lidinoid structure lie between the above two structures. The simulation results are in good agreement with the experimental data, which verifies the prediction capability of the numerical model for deformation and failure behaviors.
(2) The pore characteristics of different TPMS topologies exert a significant influence on acoustic performance, showing a consistent variation trend with mechanical properties. The sound absorption coefficient and sound insulation value of all three structures increase with rising frequency. The Diamond structure achieves a peak sound absorption coefficient close to 0.6 and a sound insulation value of approximately 4.3 dB in the high-frequency range, which are remarkably superior to those of the Lidinoid and Gyroid structures. This is closely related to its complex internal pore channel structure and efficient viscous dissipation mechanism of sound waves.
(3) Synergistic optimization of the mechano-acoustic performance of TPMS porous structures can be realized through topological regulation. Under identical fabrication conditions, the Diamond TPMS with highly symmetrical skeletal distribution achieves superior comprehensive performance balancing high load-bearing, excellent energy absorption and prominent high-frequency noise reduction compared with Gyroid and Lidinoid. Its uniform truss layout enables homogeneous stress dispersion to avoid local stress concentration for improved mechanical reliability, while the regularly distributed interconnected pores lengthen sound transmission paths and strengthen air viscous dissipation, jointly contributing to its outstanding mechano-acoustic coupling property.

This study provides theoretical references and technical support for the development of biomimetic porous structures applied in multifunctional fields including bone implants, aerospace and rail transit noise reduction protection. However, the present work is restricted to a fixed porosity and single 316L material, which constitutes certain limitations. Future research will adjust porosity, develop composite-material TPMS and optimize geometric parameters to improve low-frequency noise reduction performance.

## Figures and Tables

**Table 1:** Comparative analysis of mechanical and acoustic properties of bionic porous structures.

Structure	EA (J)	SEA (J/g)	Sound Absorption Coefficient at 6000 Hz	Sound Transmission Loss at 6000 Hz (dB)
Gyroid	503.57	25.97	0.462	3.196
Diamond	731.49	31.45	0.584	4.169
Lidinoid	633.86	27.11	0.499	3.649

## Data Availability

The original contributions presented in this study are included in the article. Further inquiries can be directed to the corresponding authors.
